# 4-Chloro­phenyl quinoline-2-carboxyl­ate

**DOI:** 10.1107/S1600536813032054

**Published:** 2013-11-30

**Authors:** E. Fazal, Manpreet Kaur, B. S. Sudha, S. Nagarajan, Jerry P. Jasinski

**Affiliations:** a Department of Chemistry, Yuvaraja’s College, Mysore 570 005, India; b Department of Studies in Chemistry, University of Mysore, Manasagangotri, Mysore 570 006, India; c P.P.S.F.T. Department, Central Food Technplogy Research institute, Mysore 570 005, India; d Department of Chemistry, Keene State College, 229 Main Street, Keene, NH 03435-2001, USA

## Abstract

In the title compound, C_16_H_10_ClNO_2_, the dihedral angle between the quinoline ring system and the benzene ring is 14.7 (5)°. The carboxyl­ate group is twisted from the mean planes of the quinoline ring system and the benzene ring by 17.7 (5) and 32.1 (4)°, respectively. In the crystal, inversion dimers are formed with the molecules linked by pairs of weak C—H⋯O inter­actions arising from an activated aromatic C atom adjacent to the C—Cl bond, generating *R*_2_^2^(14) loops.

## Related literature

For related structures, see: Fazal *et al.* (2012 ▶); Butcher *et al.* (2007 ▶); Jing & Qin (2008 ▶); Jasinski *et al.* (2010 ▶).
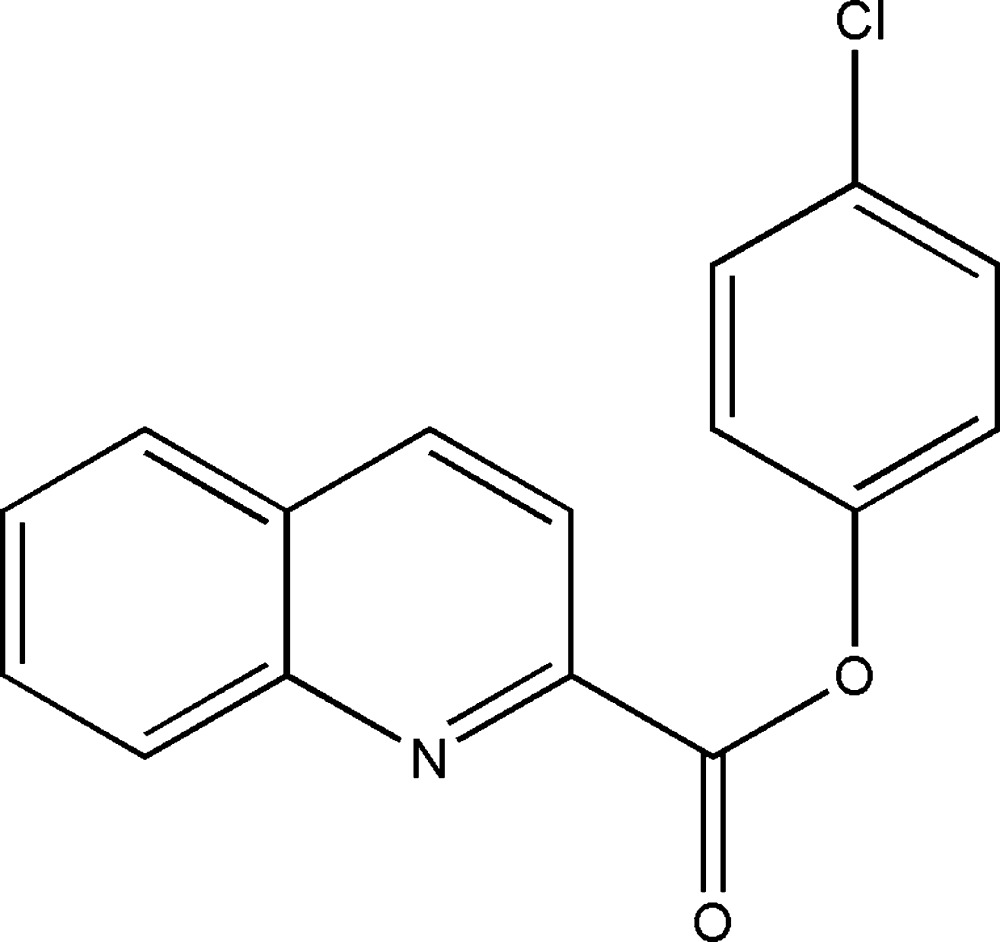


## Experimental

### 

#### Crystal data

C_16_H_10_ClNO_2_
*M* *_r_* = 283.70
Monoclinic, 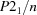

*a* = 6.38693 (18) Å
*b* = 16.8893 (5) Å
*c* = 12.2649 (4) Å
β = 103.527 (3)°
*V* = 1286.33 (6) Å^3^
*Z* = 4
Cu *K*α radiation
μ = 2.63 mm^−1^
*T* = 173 K
0.34 × 0.18 × 0.12 mm

#### Data collection

Agilent Xcalibur (Eos, Gemini) diffractometer
Absorption correction: multi-scan (*CrysAlis PRO* and *CrysAlis RED*; Agilent, 2012 ▶) *T*_min_ = 0.611, *T*_max_ = 1.000
7778 measured reflections
2502 independent reflections
2168 reflections with *I* > 2σ(*I*)
*R*_int_ = 0.032

#### Refinement

*R*[*F* ^2^ > 2σ(*F* ^2^)] = 0.039
*wR*(*F* ^2^) = 0.104
*S* = 1.05
2502 reflections
181 parameters
H-atom parameters constrained
Δρ_max_ = 0.21 e Å^−3^
Δρ_min_ = −0.25 e Å^−3^

### 

Data collection: *CrysAlis PRO* (Agilent, 2012 ▶); cell refinement: *CrysAlis PRO*; data reduction: *CrysAlis RED* (Agilent, 2012 ▶); program(s) used to solve structure: *SUPERFLIP* (Palatinus & Chapuis, 2007 ▶); program(s) used to refine structure: *SHELXL2012* (Sheldrick, 2008 ▶); molecular graphics: *OLEX2* (Dolomanov *et al.*, 2009 ▶); software used to prepare material for publication: *OLEX2*.

## Supplementary Material

Crystal structure: contains datablock(s) I. DOI: 10.1107/S1600536813032054/hb7166sup1.cif

Structure factors: contains datablock(s) I. DOI: 10.1107/S1600536813032054/hb7166Isup2.hkl

Supplementary material file. DOI: 10.1107/S1600536813032054/hb7166Isup3.cml

Additional supplementary materials:  crystallographic information; 3D view; checkCIF report

## Figures and Tables

**Table 1:** Hydrogen-bond geometry (Å, °)

*D*—H⋯*A*	*D*—H	H⋯*A*	*D*⋯*A*	*D*—H⋯*A*
C13—H13⋯O1^i^	0.93	2.42	3.250 (2)	148

Symmetry code: (i) 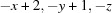.

**Fig. 1.**
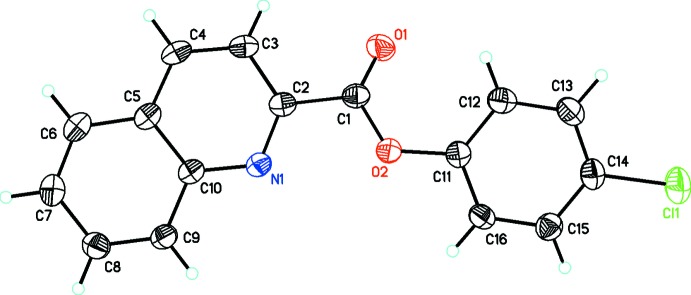
ORTEP drawing of (I) showing 50% probability displacement ellipsoids.

**Fig. 2.**
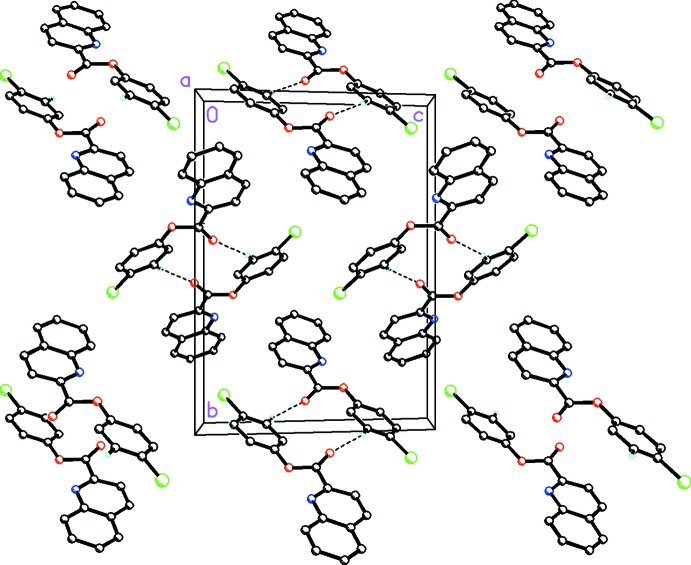
Molecular packing for (I) viewed along the a axis. Dashed lines indicate weak C13—H13···O1 interactions linking the molecules into dimers forming R22(14) loops. The remaining H atoms have been removed for clarity.

## supplementary crystallographic information

### 1. Comment

The crystal structures of 4-methylphenyl quinoline-2-carboxylate (Fazal *et al.*,2012), 1-(quinolin-2-yl)ethanone (Butcher *et al.*, 2007) and methyl quinoline-2-carboxylate (Jing & Qin, 2008) and the synthesis, crystal structures and theoretical studies of four Schiff bases derived from 4-hydrazinyl-8-(trifluoromethyl) quinoline (Jasinski *et al.*, 2010) have been reported. As part of our studies in this area, we now report the crystal structure of the title compound, (I), C_16_H_10_ClNO_2_.

The dihedral angle between the mean planes of the quinoline ring and the phenyl ring is 14.7 (5)8° (Fig. 1). The mean plane of the carboxylate group is twisted from the mean planes of the quinoline ring and phenyl ring by 17.7 (5)ånd 32.1 (4)°, respectively. In the crystal, weak C13—H13···O1 intermolecular interactions link the molecules into dimers forming R_2_^2^(14) graph set motifs (Fig. 2).

### 2. Experimental

To a mixture of 1.73 g (10 mmole) of quinaldic acid and 1.28 g (10 mmole) of 4-chlorophenol in a round-bottomed flask fitted with a reflex condenser with a drying tube was added 0.75 g (5 mmole) of phosphorous oxychloride. The mixture was heated with occasional swirling, and temperature maintained at 353-363 K. At the end of eight hours the reaction mixture was poured in to a solution of 2 g of sodium bicarbonate in 25 ml of water. The precipitated ester was collected on a filter and washed with water (yield = 2.70 g (90%). Irregula colourless crystals were obtained by recrystallization from absolute ethanol solution ( M.P.: 386 K).

### 3. Refinement

All of the H atoms were placed in their calculated positions and then refined using the riding model with Atom—H lengths of 0.93Å (CH). Isotropic displacement parameters for these atoms were set to 1.2 (CH) times *U*_eq_ of the parent atom.

### Crystal data

**Table:**

C_16_H_10_ClNO_2_	*F*(000) = 584
*M**_r_* = 283.70	*D*_x_ = 1.465 Mg m^−^^3^
Monoclinic, *P*2_1_/*n*	Cu *K*α radiation, λ = 1.54184 Å
*a* = 6.38693 (18) Å	Cell parameters from 3028 reflections
*b* = 16.8893 (5) Å	θ = 3.7–72.3°
*c* = 12.2649 (4) Å	µ = 2.63 mm^−^^1^
β = 103.527 (3)°	*T* = 173 K
*V* = 1286.33 (6) Å^3^	Irregular, colourless
*Z* = 4	0.34 × 0.18 × 0.12 mm

### Data collection

**Table:**

Agilent Xcalibur (Eos, Gemini) diffractometer	2502 independent reflections
Radiation source: Enhance (Cu) X-ray Source	2168 reflections with *I* > 2σ(*I*)
Detector resolution: 16.0416 pixels mm^-1^	*R*_int_ = 0.032
ω scans	θ_max_ = 72.5°, θ_min_ = 4.5°
Absorption correction: multi-scan (*CrysAlis PRO* and *CrysAlis RED*; Agilent, 2012)	*h* = −5→7
*T*_min_ = 0.611, *T*_max_ = 1.000	*k* = −20→20
7778 measured reflections	*l* = −15→14

### Refinement

**Table:**

Refinement on *F*^2^	Primary atom site location: structure-invariant direct methods
Least-squares matrix: full	Hydrogen site location: inferred from neighbouring sites
*R*[*F*^2^ > 2σ(*F*^2^)] = 0.039	H-atom parameters constrained
*wR*(*F*^2^) = 0.104	*w* = 1/[σ^2^(*F*_o_^2^) + (0.0544*P*)^2^ + 0.3*P*] where *P* = (*F*_o_^2^ + 2*F*_c_^2^)/3
*S* = 1.05	(Δ/σ)_max_ = 0.001
2502 reflections	Δρ_max_ = 0.21 e Å^−^^3^
181 parameters	Δρ_min_ = −0.25 e Å^−^^3^
0 restraints	

### Special details

**Table:**

Experimental. ^1^H NMR(500MHz,DMSO) δ 8.6 (1H,d, J= 8.48Hz), 8.34(1H,d, J= 8.49Hz),8.29(1H,d, J= 8.49Hz), 8.08(1H,d, J= 8.23 Hz),7.91(1H,dt, J1= 8.2Hz, J2=7, J3=1.36Hz), 7.79(1H,t, J= 7.73Hz), 7.51(2H,d, J= 8.78Hz), 7.38(2H,d, J= 8.78Hz)
Geometry. All esds (except the esd in the dihedral angle between two l.s. planes) are estimated using the full covariance matrix. The cell esds are taken into account individually in the estimation of esds in distances, angles and torsion angles; correlations between esds in cell parameters are only used when they are defined by crystal symmetry. An approximate (isotropic) treatment of cell esds is used for estimating esds involving l.s. planes.

### Fractional atomic coordinates and isotropic or equivalent isotropic displacement parameters (Å^2^)

**Table:**

	*x*	*y*	*z*	*U*_iso_*/*U*_eq_	
Cl1	1.24130 (8)	0.40648 (3)	0.40342 (4)	0.04923 (18)	
O1	0.61750 (19)	0.56656 (7)	−0.05303 (10)	0.0362 (3)	
O2	0.57559 (18)	0.60496 (7)	0.11776 (9)	0.0291 (3)	
N1	0.2144 (2)	0.68008 (8)	0.02285 (11)	0.0255 (3)	
C1	0.5257 (3)	0.60419 (9)	0.00414 (13)	0.0271 (3)	
C2	0.3381 (3)	0.65748 (9)	−0.04352 (13)	0.0257 (3)	
C3	0.3074 (3)	0.67937 (10)	−0.15754 (13)	0.0299 (4)	
H3	0.3984	0.6606	−0.2008	0.036*	
C4	0.1399 (3)	0.72894 (10)	−0.20223 (13)	0.0316 (4)	
H4	0.1179	0.7457	−0.2763	0.038*	
C5	0.0005 (3)	0.75454 (9)	−0.13540 (13)	0.0268 (3)	
C6	−0.1776 (3)	0.80535 (10)	−0.17629 (14)	0.0317 (4)	
H6	−0.2050	0.8240	−0.2496	0.038*	
C7	−0.3082 (3)	0.82679 (10)	−0.10810 (15)	0.0333 (4)	
H7	−0.4250	0.8600	−0.1353	0.040*	
C8	−0.2682 (3)	0.79909 (10)	0.00373 (14)	0.0313 (4)	
H8	−0.3602	0.8138	0.0489	0.038*	
C9	−0.0962 (3)	0.75108 (9)	0.04632 (13)	0.0285 (4)	
H9	−0.0708	0.7337	0.1202	0.034*	
C10	0.0434 (3)	0.72779 (9)	−0.02247 (12)	0.0250 (3)	
C11	0.7396 (3)	0.55652 (9)	0.17935 (13)	0.0266 (3)	
C12	0.9292 (3)	0.53939 (10)	0.14730 (14)	0.0312 (4)	
H12	0.9532	0.5588	0.0803	0.037*	
C13	1.0823 (3)	0.49268 (10)	0.21764 (15)	0.0328 (4)	
H13	1.2095	0.4797	0.1973	0.039*	
C14	1.0458 (3)	0.46548 (10)	0.31740 (15)	0.0314 (4)	
C15	0.8591 (3)	0.48398 (10)	0.35058 (14)	0.0314 (4)	
H15	0.8375	0.4659	0.4187	0.038*	
C16	0.7051 (3)	0.52997 (10)	0.28040 (14)	0.0284 (3)	
H16	0.5784	0.5430	0.3012	0.034*	

### Atomic displacement parameters (Å^2^)

**Table:**

	*U*^11^	*U*^22^	*U*^33^	*U*^12^	*U*^13^	*U*^23^
Cl1	0.0401 (3)	0.0515 (3)	0.0530 (3)	0.0174 (2)	0.0047 (2)	0.0118 (2)
O1	0.0361 (7)	0.0436 (7)	0.0297 (6)	0.0062 (5)	0.0094 (5)	−0.0075 (5)
O2	0.0313 (6)	0.0318 (6)	0.0243 (6)	0.0059 (5)	0.0068 (4)	−0.0011 (4)
N1	0.0294 (7)	0.0249 (6)	0.0221 (6)	−0.0011 (5)	0.0056 (5)	−0.0001 (5)
C1	0.0294 (8)	0.0276 (8)	0.0250 (8)	−0.0035 (6)	0.0076 (6)	−0.0032 (6)
C2	0.0282 (8)	0.0252 (8)	0.0242 (7)	−0.0038 (6)	0.0069 (6)	−0.0025 (6)
C3	0.0339 (9)	0.0343 (9)	0.0236 (8)	−0.0026 (7)	0.0110 (7)	−0.0024 (6)
C4	0.0400 (10)	0.0358 (9)	0.0192 (7)	−0.0041 (7)	0.0075 (7)	0.0021 (6)
C5	0.0305 (8)	0.0254 (8)	0.0230 (7)	−0.0055 (6)	0.0031 (6)	0.0002 (6)
C6	0.0350 (9)	0.0313 (8)	0.0262 (8)	−0.0041 (7)	0.0018 (7)	0.0045 (6)
C7	0.0307 (9)	0.0291 (8)	0.0377 (9)	0.0017 (7)	0.0033 (7)	0.0038 (7)
C8	0.0323 (9)	0.0297 (8)	0.0338 (9)	−0.0002 (7)	0.0116 (7)	−0.0007 (7)
C9	0.0334 (9)	0.0286 (8)	0.0239 (7)	−0.0005 (6)	0.0074 (6)	0.0014 (6)
C10	0.0286 (8)	0.0234 (7)	0.0224 (7)	−0.0038 (6)	0.0047 (6)	−0.0006 (6)
C11	0.0263 (8)	0.0250 (8)	0.0273 (8)	0.0001 (6)	0.0042 (6)	−0.0024 (6)
C12	0.0313 (9)	0.0342 (9)	0.0296 (8)	−0.0022 (7)	0.0104 (7)	−0.0017 (7)
C13	0.0264 (9)	0.0360 (9)	0.0370 (9)	0.0020 (7)	0.0093 (7)	−0.0053 (7)
C14	0.0281 (9)	0.0274 (8)	0.0359 (9)	0.0032 (6)	0.0020 (7)	−0.0009 (7)
C15	0.0318 (9)	0.0316 (8)	0.0305 (8)	−0.0010 (7)	0.0068 (7)	0.0020 (7)
C16	0.0249 (8)	0.0312 (8)	0.0304 (8)	0.0004 (6)	0.0093 (6)	−0.0025 (6)

### Geometric parameters (Å, º)

**Table:**

Cl1—C14	1.7459 (17)	C7—H7	0.9300
O1—C1	1.1963 (19)	C7—C8	1.415 (2)
O2—C1	1.3549 (19)	C8—H8	0.9300
O2—C11	1.4025 (19)	C8—C9	1.367 (2)
N1—C2	1.317 (2)	C9—H9	0.9300
N1—C10	1.366 (2)	C9—C10	1.419 (2)
C1—C2	1.503 (2)	C11—C12	1.388 (2)
C2—C3	1.415 (2)	C11—C16	1.383 (2)
C3—H3	0.9300	C12—H12	0.9300
C3—C4	1.368 (2)	C12—C13	1.388 (2)
C4—H4	0.9300	C13—H13	0.9300
C4—C5	1.411 (2)	C13—C14	1.377 (2)
C5—C6	1.420 (2)	C14—C15	1.383 (2)
C5—C10	1.421 (2)	C15—H15	0.9300
C6—H6	0.9300	C15—C16	1.384 (2)
C6—C7	1.361 (3)	C16—H16	0.9300
			
C1—O2—C11	120.97 (12)	C9—C8—H8	119.6
C2—N1—C10	117.23 (13)	C8—C9—H9	120.1
O1—C1—O2	125.33 (15)	C8—C9—C10	119.85 (15)
O1—C1—C2	123.05 (15)	C10—C9—H9	120.1
O2—C1—C2	111.61 (13)	N1—C10—C5	122.51 (14)
N1—C2—C1	118.18 (14)	N1—C10—C9	118.37 (14)
N1—C2—C3	124.79 (15)	C9—C10—C5	119.12 (15)
C3—C2—C1	117.03 (14)	C12—C11—O2	124.07 (14)
C2—C3—H3	121.0	C16—C11—O2	114.59 (13)
C4—C3—C2	118.07 (15)	C16—C11—C12	121.23 (15)
C4—C3—H3	121.0	C11—C12—H12	120.8
C3—C4—H4	120.2	C11—C12—C13	118.46 (15)
C3—C4—C5	119.67 (14)	C13—C12—H12	120.8
C5—C4—H4	120.2	C12—C13—H13	120.0
C4—C5—C6	122.82 (15)	C14—C13—C12	120.06 (15)
C4—C5—C10	117.69 (15)	C14—C13—H13	120.0
C6—C5—C10	119.49 (15)	C13—C14—Cl1	118.80 (13)
C5—C6—H6	120.0	C13—C14—C15	121.52 (16)
C7—C6—C5	119.96 (15)	C15—C14—Cl1	119.68 (14)
C7—C6—H6	120.0	C14—C15—H15	120.6
C6—C7—H7	119.7	C14—C15—C16	118.71 (16)
C6—C7—C8	120.66 (16)	C16—C15—H15	120.6
C8—C7—H7	119.7	C11—C16—C15	119.99 (15)
C7—C8—H8	119.6	C11—C16—H16	120.0
C9—C8—C7	120.89 (16)	C15—C16—H16	120.0
			
Cl1—C14—C15—C16	−179.15 (12)	C5—C6—C7—C8	0.2 (3)
O1—C1—C2—N1	162.83 (16)	C6—C5—C10—N1	−178.71 (14)
O1—C1—C2—C3	−16.9 (2)	C6—C5—C10—C9	1.5 (2)
O2—C1—C2—N1	−17.4 (2)	C6—C7—C8—C9	0.8 (3)
O2—C1—C2—C3	162.86 (14)	C7—C8—C9—C10	−0.7 (2)
O2—C11—C12—C13	177.89 (14)	C8—C9—C10—N1	179.69 (14)
O2—C11—C16—C15	−177.73 (14)	C8—C9—C10—C5	−0.5 (2)
N1—C2—C3—C4	1.4 (2)	C10—N1—C2—C1	−179.29 (13)
C1—O2—C11—C12	36.5 (2)	C10—N1—C2—C3	0.4 (2)
C1—O2—C11—C16	−147.26 (15)	C10—C5—C6—C7	−1.3 (2)
C1—C2—C3—C4	−178.90 (14)	C11—O2—C1—O1	−3.2 (2)
C2—N1—C10—C5	−1.7 (2)	C11—O2—C1—C2	177.06 (13)
C2—N1—C10—C9	178.15 (14)	C11—C12—C13—C14	−1.0 (2)
C2—C3—C4—C5	−1.9 (2)	C12—C11—C16—C15	−1.4 (2)
C3—C4—C5—C6	−179.43 (15)	C12—C13—C14—Cl1	179.69 (13)
C3—C4—C5—C10	0.7 (2)	C12—C13—C14—C15	−0.4 (3)
C4—C5—C6—C7	178.84 (16)	C13—C14—C15—C16	1.0 (3)
C4—C5—C10—N1	1.1 (2)	C14—C15—C16—C11	−0.1 (2)
C4—C5—C10—C9	−178.68 (14)	C16—C11—C12—C13	1.9 (2)

### Hydrogen-bond geometry (Å, º)

**Table:**

*D*—H···*A*	*D*—H	H···*A*	*D*···*A*	*D*—H···*A*
C13—H13···O1^i^	0.93	2.42	3.250 (2)	148

Symmetry code: (i) −*x*+2, −*y*+1, −*z*.
